# INTEREST GROUP SESSION—AGING WORKFORCE: WORK AND PRODUCTIVE AGING: OPPORTUNITIES AND CHALLENGES

**DOI:** 10.1093/geroni/igz038.1369

**Published:** 2019-11-08

**Authors:** Jim Emerman, Cal J Halvorsen, Jim Emerman

**Affiliations:** 1 Encore.org, San Francisco, California, United States; 2 Boston College School of Social Work, Chestnut Hill, Massachusetts, United States

## Abstract

With much of the world experiencing population aging and a strong need—and desire—among many approaching later life to work longer than past norms, individuals and institutions are experimenting with new ways of working. Yet given the complexities of navigating the work environment in later life, including aspects of cumulative (dis)advantage that help or hinder one’s work prospects, the pull to socially impactful work in the nonprofit sector, and the day-to-day experience of such work in later life, outcomes from this work can vary. Consequently, this symposium will focus on the challenges and opportunities of working longer and their relevance to a productive aging model. The first paper will provide a framework for engaging in the conversation on productive engagement in later life. It will give particular consideration to older workers with lower levels of socioeconomic status in OECD countries. The second paper will discuss results from more than 1,400 surveys of fellows and organizational hosts that have participated in the Encore Fellowships Network, which matches mid- and late-career workers (typically corporate retirees) to non-profit organizations seeking their skills and experience. The third and final paper will reveal findings from an experience sampling methods study of two groups of older adults over the age of 60: founders or leaders of social purpose organizations, and older volunteers. We will conclude by facilitating a discussion on ideas for future scholarship on longer working lives, with particular emphasis on individuals with less advantage as well as those pursuing social purpose work.

